# Construction of a Prognostic Model Based on Cuproptosis-Related lncRNA Signatures in Pancreatic Cancer

**DOI:** 10.1155/2022/4661929

**Published:** 2022-11-11

**Authors:** Wenkai Jiang, Yan Du, Wenlong Zhang, Wence Zhou

**Affiliations:** The First Clinical Medical College of Lanzhou University, No. 222, Tianshui Road (South), Chengguan, Lanzhou, Gansu 730000, China

## Abstract

**Aim:**

The aim of this study is to identify cuproptosis-related lncRNAs and construct a prognostic model for pancreatic cancer patients for clinical use.

**Methods:**

The expression profile of lncRNAs was downloaded from The Cancer Genome Atlas database, and cuproptosis-related lncRNAs were identified. The prognostic cuproptosis-related lncRNAs were obtained and used to establish and validate a prognostic risk score model in pancreatic cancer.

**Results:**

In total, 181 cuproptosis-related lncRNAs were obtained. The prognostic risk score model was constructed based on five lncRNAs (AC025257.1, TRAM2-AS1, AC091057.1, LINC01963, and MALAT1). Patients were assigned to two groups according to the median risk score. Kaplan–Meier survival curves showed that the difference in the prognosis between the high- and low-risk groups was statistically significant. Multivariate Cox analysis showed that our risk score was an independent risk factor for pancreatic cancer patients. Receiver operator characteristic curves revealed that the cuproptosis-related lncRNA model can effectively predict the prognosis of pancreatic cancer. The principal component analysis showed a difference between the high- and low-risk groups intuitively. Functional enrichment analysis showed that different genes were involved in cancer-related pathways in patients in the high- and low-risk groups.

**Conclusion:**

The risk model based on five prognostic cuproptosis-related lncRNAs can well predict the prognosis of pancreatic cancer patients. Cuproptosis-related lncRNAs could be potential biomarkers for pancreatic cancer diagnosis and treatment.

## 1. Introduction

Pancreatic cancer is the most common malignant tumor of the pancreas. Despite recent advances in surgery, chemotherapy, and targeted therapy, the 5-year survival rate of pancreatic cancer patients is only 9% [1, 2]. Approximately 80% of patients are not diagnosed until an advanced stage, which limits treatment options [3]. Therefore, the identification of diagnostic and prognostic biomarkers is crucial for patients with pancreatic cancer.

Cell death-related biomarkers can predict prognosis in patients with pancreatic cancer, including single-gene diagnosis and multigene risk models [4, 5]. Recently, scientists have proposed a new type of cell death called cuproptosis [6]. Cuproptosis is a copper-dependent and regulated cell death mechanism that is different from other known cell death regulation mechanisms. Copper ions directly bind to lipoacylated components in the tricarboxylic acid cycle, leading to abnormal aggregation of lipoacylated proteins and loss of Fe-S cluster proteins, resulting in toxic protein stress and ultimately mediating cell death [6]. Copper is an essential substance in organisms and is related to the growth and metastasis of tumor cells [7]. The serum copper concentration of patients with pancreatic cancer is significantly higher than that of normal patients, and blocking the absorption of copper can inhibit the progression of pancreatic cancer [8, 9].

Long noncoding RNAs (lncRNAs) are RNAs with a length of more than 200 nt that can regulate transcription and translation, participate in intracellular signaling pathways, and regulate substance metabolism [10, 11]. LncRNAs play an important regulatory role in various diseases, especially inflammatory, viral, and cancer diseases [12–14]. In pancreatic cancer tissues and cell lines, the expression of the lncRNA SNHG16 is significantly increased, which is associated with a poor prognosis of pancreatic cancer patients, and SNHG16 knockout can inhibit the proliferation, migration, and invasion of pancreatic cancer cells [15]. LncRNA PVT1 promotes gemcitabine resistance in pancreatic cancer and interacts with histone acetyltransferase 1 to play a synergistic role in chemotherapy resistance [16]. However, the relationship between cuproptosis-related lncRNAs and pancreatic cancer remains unclear.

In this study, we aimed to establish a new cuproptosis-related lncRNA risk model to predict the prognosis in patients with pancreatic cancer. These lncRNAs may be biomarkers of pancreatic cancer prognosis. Subsequently, various functional enrichment analyses were performed to determine the underlying mechanisms of cuproptosis-associated lncRNAs in pancreatic cancer. With this latest analysis, we can provide new strategies for the diagnosis, treatment, and prevention of pancreatic cancer.

## 2. Materials and Methods

### 2.1. Study Design

Pancreatic adenocarcinoma patients from The Cancer Genome Atlas (TCGA) database were selected as objects in this study. We conducted a cuproptosis-related lncRNA risk model to divide all patients into high-score and low-score groups, aiming to explore the prognostic value of the risk model and differences between the high- and low-risk score groups in patients with pancreatic cancer.

### 2.2. Database Download

The data from TCGA (https://cancergenome.nih.gov) and Genotype-Tissue Expression (GTEx, https://www.gtexportal.org) databases were used to analyze the expression of lncRNAs in pancreatic cancer. The differentially expressed lncRNAs were identified using the “DESeq2” package in *R* software. LncRNAs with an adjusted *P* value less than 0.05 and absolute fold-change value more than 0.6 were considered differentially expressed lncRNAs. Clinicopathological information and survival time were also downloaded. After excluding the samples with missing data, 178 pancreatic cancer samples and 171 normal samples were obtained. With the R package “caret,” 178 pancreatic cancer samples were divided into a training set (*n* = 88) and a validation set (*n* = 90).

### 2.3. Cuproptosis-Related lncRNAs

We collected the literature on and obtained 19cuproptosis-related genes (ATP7A, ATP7B, CDKN2A, DBT, DLAT, DLD, DLST, FDX1, GCSH, GLS, LIAS, LIPT1, LIPT2, MTF1, NFE2L2, NLRP3, PDHA1, PDHB, and SLC31A1). Pearson's correlation analysis was performed between cuproptosis-related genes and lncRNA expression levels to identify cuproptosis-related lncRNAs according to the correlation coefficients (>0.5) and *P* values (<0.05) in the training set.

### 2.4. Establishment of a Prognostic Risk Model

Prognostic-related cuproptosis lncRNAs were obtained through univariate Cox regression analysis (*P* value less than 0.01). Least absolute shrinkage and selection operator (LASSO) regression analysis of potential prognostic genes was carried out, and the list of prognostic genes corresponding to the best penalty parameter was obtained. The final prognostic model of lncRNAs were screened by multivariate Cox regression (*P* value less than 0.05). The risk score is as follows:

(1) $$\begin{array}{c} \text{Risk. Score}=\overset{\infty }{\underset{n=1}{\sum }}\left({\text{Coef}}_{\text{lncRNAs}}\;{\times \text{ Expression}}_{\text{LncRNAs}}\right)\text{.} \end{array}$$

All patients were divided into high-risk and low-risk groups according to the median risk score.

### 2.5. Prediction Capacity of the Risk Score Model

The Kaplan–Meier survival method was applied to evaluate the availability of the prognostic model in overall survival (OS), disease-specific survival (DSS), and progression-free survival (PFS). Risk score diagrams were used to visualize the prognostic models. Receiver operator characteristic (ROC) curves and areas under the curve (AUCs) were used to analyze the role of cuproptosis-related lncRNAs in predicting the prognosis of pancreatic cancer patients. In the entire cohort, the training set and validation set and univariate and multivariate Cox regression were used to identify the risk score as an independent predictor of the prognosis of pancreatic cancer patients. Principal component analysis (PCA) was conducted to reduce the dimensionality in the high- and low-risk groups to visualize the ability to distinguish risk scores.

### 2.6. Construction of the Nomogram

Clinicopathological features, including age, gender, grade, TNM stage (T, tumor; N, regional lymph node; M, metastasis), and prognostic risk score were taken into account to draw a nomogram using the *R* packages “survival” and “rms.” Then, a calibration curve was drawn to analyze the consistency between the estimated probability and actual probability.

### 2.7. Gene Ontology (GO) Enrichment and Kyoto Encyclopedia of Genes and Genomes (KEGG) Analyses

GO enrichment analysis is used to describe genetic products in any organism, including biological processes (BP), cellular components (CC), and molecular functions (MF) [17]. KEGG is a database for the analysis of signaling pathways from the perspective of gene and molecular networks [18]. Differentially expressed genes (DEGs) between the high score group and the low-score group for GO and KEGG enrichment analyses were carried out by using the “ClusterProfiler” package in *R* [19]. Significant enrichment was considered eligible if the *P* value was less than 0.05 and the false discovery rate was less than 0.2.

### 2.8. Statistical Analysis

Statistical analysis was performed by using the *R* software (Version 4.2.1). The baseline characteristics of patients in the training group and validation group were compared by using chi-square tests. The Wilcoxon test was used to determine the lncRNA expression difference between the tumor group and the normal group. The Pearson correlation test was used to analyze correlations. Cox regression was used to analyze the prognosis of patients by calculating hazard ratios (HRs) and their 95% confidence intervals (CIs). Univariate Cox regression analysis was used to identify the lncRNAs that had a significant relationship with the OS of pancreatic cancer patients (*P* < 0.05). Multivariate Cox regression analysis was used to construct the prognostic model by using hazard's proportionality of each lncRNA. Visualization of all data was performed using the “ggplot2” package in *R*. A *P* value less than 0.05 was considered to be statistically significant.

## 3. Results

### 3.1. Characteristics of Patients

A total of 178 pancreatic cancer patients were randomly assigned to the training set (*n* = 88) and validation set (*n* = 90). There were no differences in clinicopathological features between the two sets. The baseline characteristics of 178 pancreatic cancer patients are detailed in Table S1.

### 3.2. Identification of Differentially Expressed lncRNAs and Cuproptosis-Related lncRNAs

**Figure 1:**
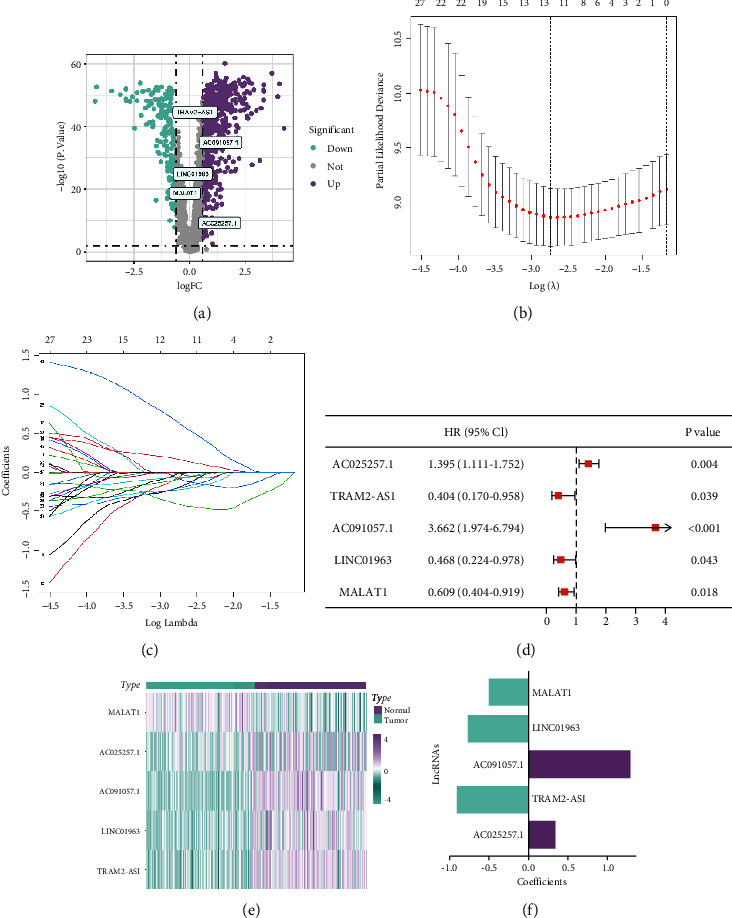
Identification of prognostic cuproptosis-related lncRNAs by LASSO regression and Cox regression analysis. (a) Volcano plot of 832 differentially expressed lncRNAs. (b, c) Selection of the optimal parameter (lambda) in the LASSO model for pancreatic cancer. (d) Forest plot of multivariate Cox regression of five lncRNAs. (e) Heatmap for the expression of five lncRNAs in pancreatic cancer tissues and normal tissues. (f) The coefficients of each lncRNA.

The expression of lncRNAs in pancreatic cancer samples and normal tissue samples was analyzed. A total of 2060 lncRNAs were obtained; among them, 832 differentially expressed lncRNAs were screened out, including 668 upregulated and 164 downregulated lncRNAs (Figure 1(a)). Correlation analysis was carried out between 832 differentially expressed lncRNAs and 19 cuproptosis-related genes. The results showed that there were 181 cuproptosis-related lncRNAs.

Univariate Cox regression analysis revealed 33 of 181 cuproptosis-related lncRNAs that were significantly related to the OS of pancreatic cancer (*P* < 0.05) (Table S2). Then, 13 lncRNAs were selected by LASSO regression analysis (Figures 1(b) and 1(c)). Finally, five lncRNAs (AC025257.1, TRAM2-AS1, AC091057.1, LINC01963, and MALAT1) were obtained by multivariate Cox regression analysis (Figure 1(d)). The relationship between the lncRNA expression and tissue type and the coefficient of each lncRNA are shown in Figures 1(e) and 1(f). The prognostic model was constructed based on these five lncRNAs in the training set.

### 3.3. Establishment and Validation of the Prognostic Model

**Figure 2:**
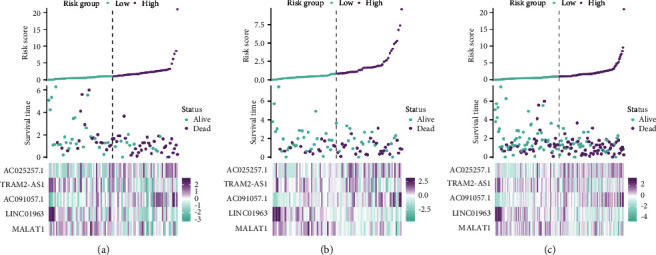
The distribution of risk score, patient survival time, patient status, and expression level of five lncRNAs for pancreatic cancer. (a) Training set. (b) Validation set. (c) Entire cohort.

The calculation of the risk score was 0.3335 ×ExpressionAC025257.1 − 0.9055 × ExpressionTRAM2-AS1 + 1.2982 × ExpressionAC091057.1 − 0.7587 × ExpressionLINC01963 − 0.4945 × ExpressionMALAT1. Each patient's risk score was calculated, and all patients were divided into high-risk and low-risk groups according to the median risk score. The relationship between survival time or mortality and risk score is shown in Figures 2(a)–2(c).

**Figure 3:**
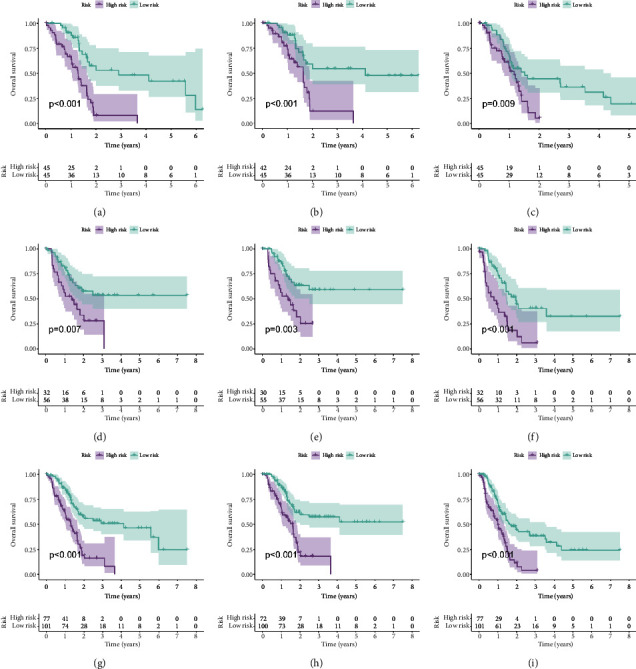
Kaplan–Meier curves of the risk score based on OS, DSS, and PFS in the three sets. (a) OS, (b) DSS, and (c) PFS in the training set. (d) OS, (e) DSS, and (f) PFS in the validation set. (g) OS, (h) DSS, and (i) PFS in the entire cohort.

In the training group, Kaplan–Meier curves showed that patients with high scores had shorter OS (HR = 3.18, 95% CI (1.80–5.62), *P* < 0.001, Figure 3(a)), poorer DSS (HR = 2.94, 95% CI (1.50–5.74), *P*=0.004, Figure 3(b)), and worse PFS (HR = 1.94, 95% CI (1.11–3.37), *P*=0.009, Figure 3(c)). To verify the accuracy of the prognostic model constructed in the training set, the risk score was applied to the validation set and the entire group. Both of them showed results similar to those of the training set (Figures 3(d)–3(i)).

**Figure 4:**
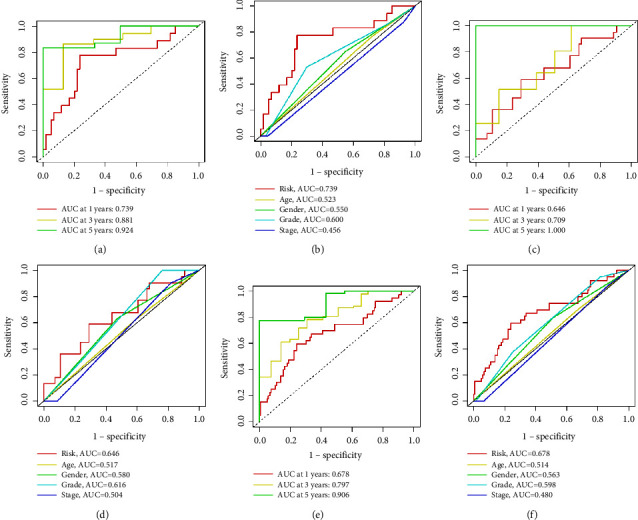
ROC analysis of the sensitivity and specificity of OS for cuproptosis-related lncRNAs in pancreatic cancer. (a, b) Training set. (c, d) Validation set. (e, f) Entire cohort.

In the ROC curves, the 1-, 3-, and 5-year AUC values of the risk score in the training set were 0.739, 0.881, and 0.924, respectively, and the prognostic accuracy of the risk score was higher than that of other clinical characteristics (age, sex, grade, and TNM stage) (Figures 4(a) and 4(b)). The AUCs for the 1-, 3-, and 5-year OS rates in the validation set were 0.646, 0.709, and 1.000, respectively, and in the entire group, they were 0.678, 0.797, and 0.906, respectively. The prognostic accuracies of the risk score were also higher than those of other clinical characteristics, indicating that the risk model is stable and efficient in predicting the prognosis of pancreatic cancer patients (Figures 4(c)–4(f)).

**Figure 5:**
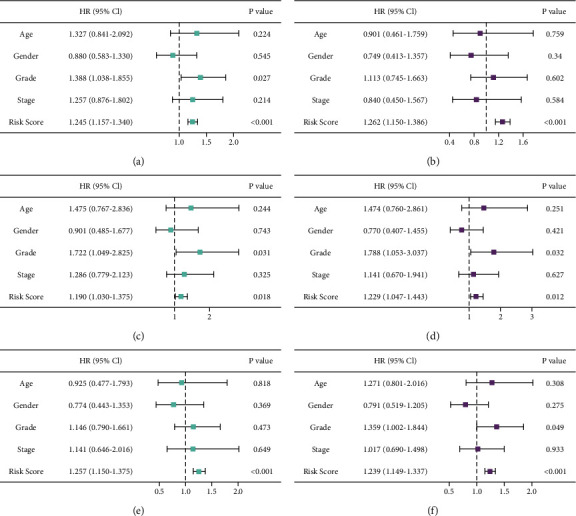
Independent prognostic analysis in the three groups. (a, b) Training set. (c, d) Validation set. (e, f) Entire cohort.

Univariate Cox regression showed that the risk score was a prognostic factor in the training set (HR = 1.245, 95% CI 1.157–1.340, *P* < 0.001, Figure 5(a)). Multivariate Cox regression analysis showed that the risk score was an independent prognostic predictor (HR = 1.262, 95% CI 1.150–1.386, *P* < 0.001, Figure 5(b)). The same results were obtained in the validation set (HR = 1.229, 95% CI 1.047–1.443, *P*=0.012, Figure 5(d)) and entire cohort (HR = 1.239, 95% CI 1.149–1.337, *P* < 0.001, Figure 5(f)).

### 3.4. PCA

**Figure 6:**
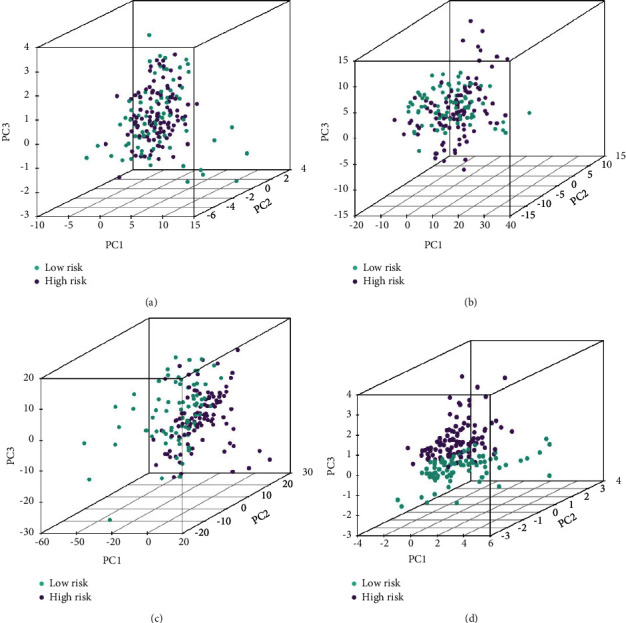
Principal component analysis (PCA) based on the five cuproptosis-related lncRNAs showed that the low-risk group and high-risk group tended to separate into two sides. (a) Cuproptosis-related genes. (b) Cuproptosis-related lncRNAs. (c) Differentially expressed lncRNAs. (d) Risk score model.

To explore the distribution of high- and low-risk groups, we performed PCA. Compared with cuproptosis-related genes, cuproptosis-related lncRNAs, and differentially expressed lncRNAs, all patients in the three cohorts could be clearly divided into two clusters based on our prognostic model (Figures 6(a)–6(d)), that is, the prognostic model was used to separate the pancreatic cancer patients into two sections, indicating that the cuproptosis status of the pancreatic cancer patients in the low-risk group was distinguishable from that in the high-risk group.

### 3.5. Nomogram Based on the Combination of Risk Score and Clinicopathology

**Figure 7:**
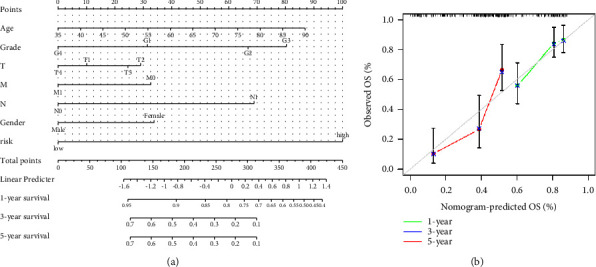
The nomogram to anticipate prognostic probabilities in pancreatic cancer. (a) Nomogram based on risk score and clinicopathological characteristics. (b) The calibration curves of the nomogram in the training set, which was used to predict the 1-, 3-, and 5-year OS.

To improve the accuracy of prognostic evaluation of pancreatic cancer patients from different levels, we established a prognostic analysis nomogram of prognostic cuproptosis-related lncRNAs and clinicopathological features to predict the survival probability at 1, 3, and 5 years based on the training set (Figure 7(a)). Verified by the calibration analysis, there was excellent consistency between the nomogram prediction and actual observation (Figure 7(b)).

### 3.6. GO and KEGG Analysis

**Figure 8:**
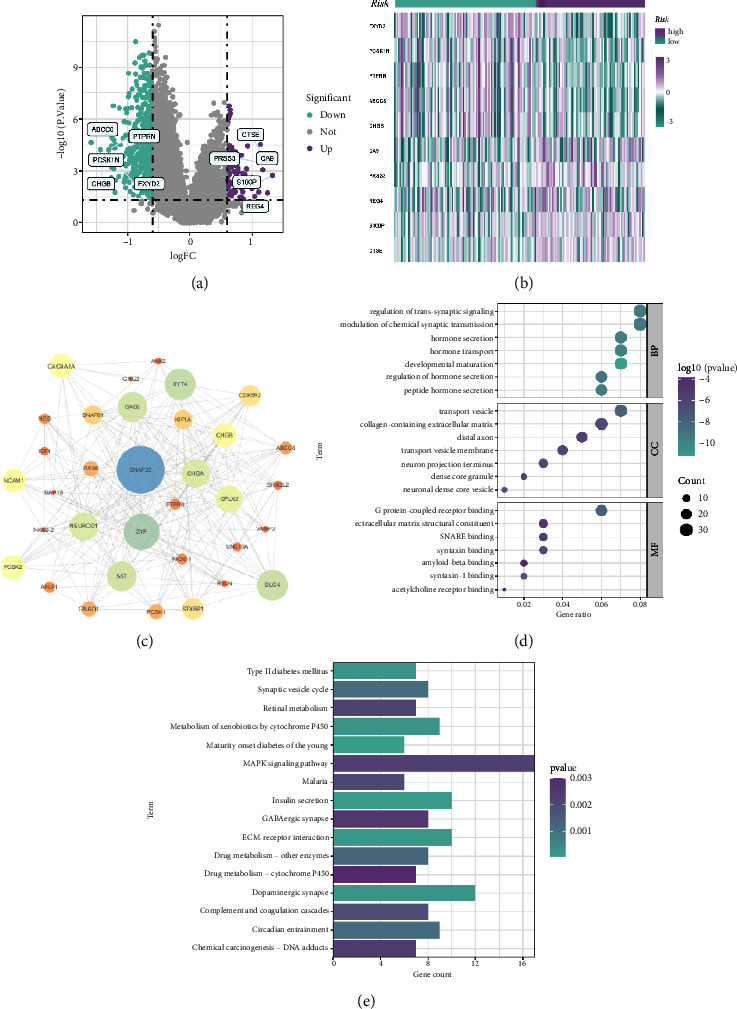
Functional enrichment analysis using GO and KEGG analysis. (a) Volcano diagram: each point represents a protein: downregulated (purple), upregulated (green), and no significant (gray). (b) Heatmap: higher expression genes are shown in purple; lower expressions genes are shown in green. (c) PPI network of the most significant difference genes between two high- and low-risk groups. (d) GO analysis. (e) KEGG analysis.

To investigate potential biological functions and pathways between the high-score group and the low-score group, we performed functional enrichment analysis of the DEGs. There were 973 DEGs between the high- and low-risk groups (Figure 8(a)). The heatmap and PPI interaction networks of the top 10 DEGs are shown in Figures 8(b) and 8(c). The GO and KEGG enrichment analyses showed that DEGs are mainly involved in many biological effects and signaling pathways (Figures 8(d) and 8(e)). For instance, BP contains regulation of transsynaptic signaling, modulation of chemical synaptic transmission, hormone transport, and peptide hormone secretion. CC contains transport vesicles, collagen-containing extracellular matrix, distal axons, and transport vesicle membranes. MF contains G protein-coupled receptor binding, chemokine activity, and extracellular matrix structural constituents. The KEGG analysis also showed significant enrichment of DEGs in cancer-related pathways, for instance, ECM-receptor interaction, cell adhesion molecules, MAPK signaling pathway, chemokine signaling pathway, and IL-17 signaling pathway.

## 4. Discussion

Cell death is required for normal development and maintenance of tissue homeostasis in multicellular organisms [20]. Currently, the most studied forms of programmed cell death include apoptosis, necrosis, ferroptosis, and autophagy [21]. Programmed cell death is closely associated with the development of cancer [22]. Cuproptosis is a new cell death mode that is different from the known cell death regulation. Since copper content and copper metabolism are associated with tumor diseases, we identified cuproptosis-related lncRNAs and constructed a risk model to predict the prognosis of pancreatic cancer patients. These lncRNAs may become novel biomarkers for pancreatic cancer, and the prediction performance of our prognostic model was good not only in the training set, but also in the validation set and entire cohort.

In this study, we found that 33 cuproptosis-related lncRNAs in TCGA-PAAD cohort were associated with pancreatic cancer survival. We also performed multivariate Cox regression analysis on prognosis-related cuproptosis lncRNAs and found that five lncRNAs, AC025257.1, TRAM2-AS1, AC091057.1, LINC01963, and MALAT1, exhibited significant prognostic value for pancreatic cancer. Gene signatures based on copper metabolism-related genes have been reported in a variety of tumor diseases, and prognostic models based on several biological functions have also been widely used in pancreatic cancer [4, 23–26]. We established a risk score to predict the prognosis of pancreatic cancer based on five cuproptosis-related lncRNAs. All patients were randomly divided into two sets (training and validation sets) in TCGA cohort. Through Kaplan–Meier survival analysis, Cox multivariate regression, risk curve analysis, ROC curve analysis, and PCA, our prognostic model can provide a reference value for evaluating the prognosis of patients with pancreatic cancer. These results demonstrated the accuracy and reliability of the prognostic model based on cuproptosis-related lncRNAs.

KRAS is one of the major oncogenes of pancreatic cancer. Searching for new biomarkers and molecular targets of pancreatic cancer is a hot research topic at present. LncRNAs play an important role in the development of pancreatic cancer, and comprehensive analysis and identification of lncRNA-related prognostic models have also been applied in pancreatic cancer [4, 27, 28]. In our prognostic model, we identified five cuproptosis-related lncRNAs that were associated with the prognosis of pancreatic cancer. MALAT1 is one of the most abundant lncRNAs in normal tissues, and emerging evidence has linked MALAT1 to lung cancer, breast cancer, prostate cancer, and pancreatic cancer [29]. In pancreatic cancer, importin 7 (a nuclear transport factor) inhibits the expression of p53 and induces the expression of MALAT1, resulting in the progression of pancreatic cancer [30]. LINC01963 is expressed at lower levels in pancreatic cancer tissues and cell lines. Upregulated expression of LINC01963 in pancreatic cancer cell lines can inhibit the cell cycle, proliferation and invasion, and promote cell apoptosis. In addition, LINC01963 negatively regulates the expression of microRNA-641 and inhibits the progression of pancreatic cancer [31]. Researchers have also identified seven lncRNAs, including AC091057.1, and generated a risk model, as well as a nomogram, that could predict the prognosis of cancer patients [32].

We established a nomogram to predict the clinical outcomes of pancreatic cancer patients. Nomograms are stable and reliable tools for quantifying individual risk and are widely used for cancer prognosis [33]. In addition to clinicopathologic features, prognostic models based on novel biomarkers can also be incorporated into nomogram models [34]. For instance, a nomogram predicted 1-, 3-, and 5-year OS rates for pancreatic cancer patients and gave a prognostic score calculated by m5C-relatedlnRNAs [27]. The combination of prognosis-associated immune genes and prognostic factors has a better prognostic value than the single application in colorectal cancer [35]. The nomogram in our study, which contains risk scores and other clinicopathologic characteristics, can effectively predict the survival in 1-, 3-, and 5-year pancreatic cancer patients.

Based on our prognostic model, all patients were divided into two cohorts (high- and low-risk score). We also performed an enrichment analysis between the two groups to further explore the efficacy of our model in determining pancreatic cancer. The DEGs in the two groups were mainly involved in the process of cancer-related signaling pathways and biological functions, including tumor microenvironment-related mechanisms and immune-related mechanisms. Pancreatic cancer progression is associated with the tumor microenvironment, in which the expression of chemokines, accumulation of extracellular matrix, and the action of cancer-promoting cytokines accelerate the growth, invasion, and metastasis of pancreatic cancer [36–38]. IL-17 can promote the progression of pancreatic cancer, and the high expression of IL-17 can activate the Notch pathway through the NF-KB pathway. The inhibition of IL-17 and the Notch pathway can enhance the therapeutic effect by inhibiting pancreatic cancer growth in vivo [39]. Studies have shown that activation of the MAPK pathway is involved in the interaction between pancreatic cancer cells and cancer-associated fibroblasts [39]. Our KEGG analysis also showed that ECM-receptor interactions and the MAPK pathway were significantly enriched in the DEGs of the high- and low-risk score groups. These results improved our understanding of the sophisticated reciprocities between pancreatic cancer and the tumor microenvironment and might identify novel therapeutic targets.

Immune checkpoints are receptors expressed by immune cells that contribute to immune escape from tumors, most commonly programmed cell death protein 1 and cytotoxic T lymphocyte-associated protein 4 [40]. In recent years, immune checkpoint blockade (ICB) has been applied in the immunotherapy of various tumor diseases [41]. However, ICB is less effective in pancreatic cancer. The tumor microenvironment of pancreatic cancer is in an immunosuppressive state, and the existence of a variety of immunosuppressive cells, immunosuppressive factors, and cellular pathways hinders the response of cancer cells to immunotherapy [42]. Moreover, angiogenesis and immunosuppression frequently occur simultaneously [42]. As we identified the clinical significance and function of the five lncRNAs, combined immunotherapy or antiangiogenic therapy with targeting cuproptosis-related lncRNAs may provide new ideas for the treatment of pancreatic cancer in the future.

There were several limitations in this study. First, the number of lncRNAs in the Gene Expression Omnibus (GEO) datasets was so small that they could not be used as the validation set. Therefore, we could only randomly divide TCGA-PAAD dataset into the training set and the validation set, which inevitably increased the bias in the study. Then, we deleted several samples that contained incomplete clinicopathological features, which caused information bias to a certain extent. Our prognostic model needs to be further validated by multicenter, large-scale clinical trials or datasets.

## 5. Conclusion

In summary, we identified five cuproptosis-related lncRNAs as potential biomarkers for pancreatic cancer. The risk model based on the five lncRNAs can well predict the prognosis of pancreatic cancer patients. Our study not only has important significance in predicting the prognosis of pancreatic cancer, but also has certain guiding significance for future research on cancer mechanisms based on cuproptosis.

## Data Availability

The original data presented in the study were collected from TCGA (https://cancergenome.nih.gov) database.

## Supplementary Materials

Supplementary Materials Supplementary Table S1: baseline characteristics of patients in the three groups. Supplementary Table S2: univariate Cox regression analysis of 181 cuproptosis-related lncRNAs.
