# Supplementary material for: Construction of a Prognostic Model Based on Cuproptosis-Related lncRNA Signatures in Pancreatic Cancer
Source: Can J Gastroenterol Hepatol. 2022 Nov 11;2022:4661929. doi: 10.1155/2022/4661929 (PMC9674419; doi:10.1155/2022/4661929)
Supplement: Supplementary Materials — Supplementary Table S1: baseline characteristics of patients in the three groups. Supplementary Table S2: univariate Cox regression analysis of 181 cuproptosis-related lncRNAs. [file 4661929.f1.docx]

| **Table S1**. Baseline characteristics of patients in three groups | | | | |
| --- | --- | --- | --- | --- |
| Characteristics | No. (%) | | | |
|  | Entire cohort (n=178) | Training set (n=90) | Validation set (n=88) | Pvalue |
| Age |  |  |  | 0.1227 |
| <=60 | 58(32.58%) | 24(26.67%) | 34(38.64%) |  |
| >60 | 120(67.42%) | 66(73.33%) | 54(61.36%) | |
| Gender |  |  |  | 0.7748 |
| Female | 80(44.94%) | 39(43.33%) | 41(46.59%) |  |
| Male | 98(55.06%) | 51(56.67%) | 47(53.41%) | |
| Histologic grade | |  |  | 0.1789 |
| G1 | 31(17.42%) | 15(16.67%) | 16(18.18%) |  |
| G2 | 95(53.37%) | 42(46.67%) | 53(60.23%) |  |
| G3 | 48(26.97%) | 30(33.33%) | 18(20.45%) |  |
| G4 | 2(1.12%) | 2(2.22%) | 0(0%) |  |
| unknown | 2(1.12%) | 1(1.11%) | 1(1.14%) |  |
| Pathologic stage | |  |  | 0.2732 |
| stage1 | 21(11.8%) | 8(8.89%) | 13(14.77%) |  |
| stage2 | 146(82.02%) | 77(85.56%) | 69(78.41%) |  |
| stage3 | 3(1.69%) | 0(0%) | 3(3.41%) |  |
| stage4 | 5(2.81%) | 3(3.33%) | 2(2.27%) |  |
| unknown | 3(1.69%) | 2(2.22%) | 1(1.14%) |  |
| T stage |  |  |  | 0.2281 |
| T1 | 7(3.93%) | 3(3.33%) | 4(4.55%) |  |
| T2 | 24(13.48%) | 9(10%) | 15(17.05%) |  |
| T3 | 142(79.78%) | 77(85.56%) | 65(73.86%) |  |
| T4 | 3(1.69%) | 0(0%) | 3(3.41%) |  |
| unknown | 2(1.12%) | 1(1.11%) | 1(1.14%) |  |
| M stage |  |  |  | 0.7746 |
| M0 | 170(95.51%) | 85(94.44%) | 85(96.59%) |  |
| M1 | 5(2.81%) | 3(3.33%) | 2(2.27%) |  |
| unknown | 3(1.69%) | 2(2.22%) | 1(1.14%) |  |
| N stage |  |  |  | 0.0606 |
| N0 | 50(28.09%) | 20(22.22%) | 30(34.09%) | |
| N1 | 123(69.1%) | 69(76.67%) | 54(61.36%) |  |
| unknown | 5(2.81%) | 1(1.11%) | 4(4.55%) |  |

**Table S2**. Univariate Cox regression analysis of 181 cuprotosis-related lncRNAs

| LncRNAs | HR | HR.95L | HR.95H | p value |
| --- | --- | --- | --- | --- |
| RNF185-AS1 | 0.938551463 | 0.720112817 | 1.223251174 | 0.638950621 |
| AL929472.2 | 0.705310889 | 0.527214401 | 0.943569541 | 0.018715917 |
| AC025257.1 | 1.214176914 | 1.022904603 | 1.441215117 | 0.02649395 |
| LINC00662 | 0.767128456 | 0.444137691 | 1.325008164 | 0.341745333 |
| CTBP1-DT | 1.001374256 | 0.672919751 | 1.490148564 | 0.994597255 |
| AL590133.2 | 0.898646084 | 0.755490248 | 1.068928138 | 0.227405588 |
| MKLN1-AS | 1.153263832 | 0.72143593 | 1.843569763 | 0.551324932 |
| AC023509.1 | 0.831478543 | 0.536265497 | 1.289205759 | 0.409519655 |
| AC007192.2 | 0.731100164 | 0.556672663 | 0.960182681 | 0.024314031 |
| MSC-AS1 | 1.099033759 | 0.818380565 | 1.475933391 | 0.530202694 |
| AC005921.2 | 0.818308413 | 0.649061828 | 1.031687012 | 0.089868143 |
| AL137058.2 | 0.731384721 | 0.377447906 | 1.417211758 | 0.354012568 |
| AL031600.3 | 0.81018698 | 0.666972509 | 0.98415292 | 0.033928586 |
| AC005288.1 | 1.048464914 | 0.65743512 | 1.672071728 | 0.842465707 |
| EIF3J-DT | 0.832309017 | 0.527143607 | 1.314135825 | 0.430888239 |
| AC005332.6 | 0.785223277 | 0.498972753 | 1.235689905 | 0.295947761 |
| NORAD | 1.071254344 | 0.709920029 | 1.616500201 | 0.742994885 |
| FTX | 1.046424482 | 0.617381711 | 1.773625906 | 0.866140899 |
| AL122035.1 | 0.740983033 | 0.400727434 | 1.370147906 | 0.339151478 |
| AC012615.2 | 0.798622328 | 0.660700665 | 0.965335222 | 0.020088587 |
| AC104116.1 | 0.813735775 | 0.654245825 | 1.012105675 | 0.064046628 |
| AC018557.1 | 0.77149017 | 0.63738172 | 0.933815739 | 0.007749277 |
| AC087393.2 | 0.81329835 | 0.698517675 | 0.946939827 | 0.007760104 |
| AC022509.3 | 1.192269527 | 0.746597454 | 1.903980006 | 0.461517468 |
| DHRS4-AS1 | 0.871006748 | 0.568428648 | 1.334649051 | 0.525916842 |
| BAALC-AS1 | 1.077249144 | 0.604079754 | 1.921047199 | 0.80094694 |
| AC138035.1 | 0.833324045 | 0.571614767 | 1.214854837 | 0.343115932 |
| DUBR | 0.937185824 | 0.533803418 | 1.645394613 | 0.821277253 |
| AC011603.2 | 0.916999205 | 0.82451475 | 1.019857488 | 0.110163639 |
| AC092338.1 | 1.088804778 | 0.739003496 | 1.604181646 | 0.666978735 |
| AL451085.2 | 0.60389615 | 0.409696322 | 0.890148484 | 0.010840224 |
| TRAM2-AS1 | 0.570759516 | 0.345777399 | 0.94212758 | 0.02830003 |
| AC018647.2 | 0.910771722 | 0.484878393 | 1.710748799 | 0.771367505 |
| OIP5-AS1 | 1.55465624 | 0.907532977 | 2.663215647 | 0.108124827 |
| AC145207.2 | 0.955128725 | 0.818993256 | 1.113893031 | 0.558439778 |
| AL358781.1 | 0.796480145 | 0.66520868 | 0.95365656 | 0.013272983 |
| HCG18 | 0.746422606 | 0.482761771 | 1.154082074 | 0.188369675 |
| NNT-AS1 | 0.7972557 | 0.447296962 | 1.421017142 | 0.442260461 |
| AC099329.1 | 1.156911776 | 0.994003006 | 1.34651993 | 0.059797328 |
| HCG11 | 1.018306505 | 0.700513872 | 1.480267814 | 0.924276973 |
| AC108449.3 | 0.896532579 | 0.759727053 | 1.057972943 | 0.196053814 |
| PAXIP1-AS2 | 1.274002151 | 0.802798951 | 2.021778278 | 0.304065728 |
| AL450306.1 | 0.847274967 | 0.687010153 | 1.044926143 | 0.121340008 |
| AC005562.1 | 0.8954656 | 0.457184366 | 1.753906523 | 0.74752512 |
| AL731577.2 | 1.415880315 | 0.934025653 | 2.146319065 | 0.101337631 |
| FGD5-AS1 | 1.002496947 | 0.679309401 | 1.479443869 | 0.9899792 |
| LINC01184 | 0.734423828 | 0.421516837 | 1.279612846 | 0.275885216 |
| AC005034.3 | 1.422028343 | 0.668302425 | 3.025822638 | 0.360777927 |
| FAM111A-DT | 1.488935037 | 0.848704797 | 2.612130334 | 0.165145848 |
| AP001189.3 | 0.884183719 | 0.577085774 | 1.354704768 | 0.571783688 |
| SLC8A1-AS1 | 0.83603636 | 0.619372339 | 1.128492106 | 0.241951413 |
| ARAP1-AS1 | 0.906006071 | 0.760166763 | 1.079824904 | 0.270321469 |
| LINC01560 | 0.935517239 | 0.486555733 | 1.798750783 | 0.841608399 |
| AC090559.1 | 1.026371302 | 0.729188082 | 1.444672608 | 0.881366884 |
| AC091053.1 | 0.878335514 | 0.757128734 | 1.018945974 | 0.086852674 |
| OTUD6B-AS1 | 0.790743577 | 0.535593974 | 1.167442941 | 0.237552906 |
| AP001107.2 | 0.913043472 | 0.789117609 | 1.056431098 | 0.221576539 |
| LINC02038 | 1.05956477 | 0.764909023 | 1.467726838 | 0.727835697 |
| AP003068.1 | 0.966758767 | 0.777642268 | 1.201866914 | 0.760835164 |
| AL136131.3 | 1.006144619 | 0.805086298 | 1.257414263 | 0.95704926 |
| AC021218.1 | 1.126206074 | 0.899231209 | 1.410471643 | 0.300661574 |
| MIR31HG | 1.262351575 | 0.961573095 | 1.657213068 | 0.093390855 |
| AC022034.1 | 1.32774615 | 0.941960983 | 1.871531699 | 0.105538093 |
| AC007620.2 | 1.007147534 | 0.773802032 | 1.310860029 | 0.957761137 |
| AC018521.2 | 0.845896958 | 0.671684723 | 1.065293938 | 0.154913176 |
| AL671710.1 | 0.580306327 | 0.346237957 | 0.972612696 | 0.038889457 |
| AC015922.2 | 0.844644128 | 0.54028993 | 1.320446046 | 0.45891836 |
| AC011472.3 | 0.976159755 | 0.708312326 | 1.345293357 | 0.882779951 |
| LINC01094 | 1.185292004 | 0.851832954 | 1.649287139 | 0.31319905 |
| CARD8-AS1 | 0.904675708 | 0.569718339 | 1.436566249 | 0.671132959 |
| ABALON | 1.547643684 | 0.902464381 | 2.654067042 | 0.112504943 |
| AC080112.1 | 1.237806497 | 0.699538158 | 2.190252107 | 0.463734748 |
| AC124319.1 | 1.0954701 | 0.778368466 | 1.541756625 | 0.601000465 |
| AC099850.3 | 1.466333109 | 0.954025222 | 2.253748368 | 0.080923362 |
| AL121603.2 | 1.081362302 | 0.566717194 | 2.063365011 | 0.812438298 |
| RNF216P1 | 0.688488098 | 0.422447913 | 1.122069361 | 0.134186549 |
| ZEB1-AS1 | 0.722809968 | 0.483515552 | 1.080532463 | 0.113559689 |
| AC115618.2 | 1.274060721 | 0.760182658 | 2.135316694 | 0.357949274 |
| AP001372.2 | 0.926768838 | 0.589409002 | 1.457223213 | 0.741892903 |
| AC091057.1 | 1.912116903 | 1.161601691 | 3.14754281 | 0.010801978 |
| AC010655.4 | 1.487080308 | 0.983674152 | 2.248110147 | 0.059849672 |
| NUTM2B-AS1 | 0.766659362 | 0.445924971 | 1.31808402 | 0.336525059 |
| AL160408.2 | 0.854471448 | 0.565222887 | 1.291740786 | 0.455735138 |
| MIR22HG | 0.618268532 | 0.419971905 | 0.910194166 | 0.014815947 |
| MAP4K3-DT | 0.774580459 | 0.44622376 | 1.344560605 | 0.363995099 |
| AC026471.1 | 0.574961051 | 0.27857392 | 1.186687578 | 0.134395601 |
| AL138478.1 | 0.686078604 | 0.460210296 | 1.022801653 | 0.064414303 |
| AC009088.3 | 0.94650605 | 0.809582206 | 1.10658769 | 0.490451807 |
| AL133355.1 | 1.02808503 | 0.563580925 | 1.875434 | 0.92804393 |
| THAP9-AS1 | 1.052769396 | 0.731049302 | 1.516072033 | 0.782268414 |
| AC007383.2 | 0.612656002 | 0.408846449 | 0.918064415 | 0.017585924 |
| AL118506.1 | 0.589452808 | 0.387842766 | 0.895864621 | 0.013329142 |
| AC008894.2 | 1.136910713 | 0.626215749 | 2.064090485 | 0.673243002 |
| AL583722.2 | 0.883051 | 0.549271861 | 1.419659597 | 0.607659214 |
| NOP14-AS1 | 0.857533489 | 0.541354885 | 1.358376372 | 0.512542702 |
| TNFRSF10A-AS1 | 1.236291595 | 0.8688989 | 1.759027325 | 0.23842974 |
| AC119427.1 | 1.13738204 | 0.826648118 | 1.564919676 | 0.429141131 |
| MIR4435-2HG | 1.146884758 | 0.859089088 | 1.531092253 | 0.352541278 |
| AC093297.2 | 0.65201546 | 0.382375976 | 1.111796207 | 0.11624137 |
| LINC01963 | 0.588025128 | 0.356821683 | 0.969037389 | 0.0372176 |
| LINC00847 | 0.705910249 | 0.465077971 | 1.071453199 | 0.101881685 |
| ZNF667-AS1 | 0.753260545 | 0.564768226 | 1.004662485 | 0.053816946 |
| AC006504.5 | 0.667297564 | 0.399005775 | 1.115988955 | 0.123142651 |
| ZNF561-AS1 | 0.892305563 | 0.551779695 | 1.442983904 | 0.642192911 |
| AC125257.1 | 0.502768188 | 0.295638737 | 0.855016001 | 0.011144765 |
| AC010478.1 | 0.631591517 | 0.400092035 | 0.997040205 | 0.048530978 |
| MAGI2-AS3 | 0.93010152 | 0.709874701 | 1.218650048 | 0.599160715 |
| SNHG14 | 0.693635727 | 0.518839173 | 0.927321118 | 0.013537432 |
| AC009318.2 | 0.689937878 | 0.305367263 | 1.558825499 | 0.372135948 |
| DLEU1 | 0.873594791 | 0.546041139 | 1.397638026 | 0.572999388 |
| AL050341.2 | 0.518510925 | 0.325962762 | 0.824798445 | 0.005549526 |
| CASC2 | 1.072957318 | 0.572232789 | 2.011834042 | 0.826218178 |
| CD27-AS1 | 0.694015705 | 0.467787058 | 1.029651827 | 0.069557399 |
| AC004812.2 | 0.430629434 | 0.235130518 | 0.788675633 | 0.006354373 |
| MAN1B1-DT | 0.489839021 | 0.249242185 | 0.962687222 | 0.038426715 |
| LINC01003 | 0.34357361 | 0.191975823 | 0.61488381 | 0.000321131 |
| AL118558.3 | 0.377660299 | 0.211335987 | 0.674884118 | 0.001010872 |
| SNHG11 | 0.623260967 | 0.384561355 | 1.010122906 | 0.054974069 |
| ILF3-DT | 0.533956785 | 0.315928217 | 0.902451357 | 0.019114303 |
| AC073508.3 | 0.68352735 | 0.373889355 | 1.249593316 | 0.216423561 |
| UBL7-AS1 | 1.160612073 | 0.507913745 | 2.652065233 | 0.723892781 |
| LINC00909 | 0.635685601 | 0.418322273 | 0.965992511 | 0.033835954 |
| NIFK-AS1 | 0.475932139 | 0.2628816 | 0.861647987 | 0.014219998 |
| LINC01091 | 0.60882212 | 0.407214225 | 0.91024417 | 0.015595022 |
| AC002467.1 | 0.881963591 | 0.460365338 | 1.689657564 | 0.704938153 |
| SCAMP1-AS1 | 0.551446485 | 0.330229111 | 0.920855297 | 0.022898162 |
| AC009779.2 | 0.502597437 | 0.281768915 | 0.896494149 | 0.019805066 |
| UBA6-AS1 | 0.693454511 | 0.398350106 | 1.207177184 | 0.195572749 |
| AC093726.1 | 0.961534571 | 0.555592436 | 1.664077248 | 0.888530719 |
| AC092747.4 | 0.68151177 | 0.377490805 | 1.230383063 | 0.203327406 |
| WAC-AS1 | 0.488664941 | 0.294568966 | 0.810653707 | 0.005557754 |
| AP000787.1 | 0.637871092 | 0.327964606 | 1.240620243 | 0.185267525 |
| MALAT1 | 0.70962696 | 0.525646779 | 0.958001537 | 0.025079587 |
| AL138756.1 | 0.497379907 | 0.261136561 | 0.947346368 | 0.033627951 |
| MIR100HG | 0.902926697 | 0.686537876 | 1.187518778 | 0.465090844 |
| AC026356.1 | 1.158467822 | 0.648583458 | 2.069198155 | 0.61916948 |
| AC083798.2 | 1.285318076 | 0.737064917 | 2.241379992 | 0.376324003 |
| AC025262.3 | 1.027177612 | 0.781348474 | 1.350349916 | 0.847642646 |
| NCK1-DT | 1.590832318 | 0.902386181 | 2.804506006 | 0.10851681 |
| AC080038.1 | 1.104759679 | 0.897356327 | 1.360099564 | 0.347679863 |
| AC009237.14 | 0.89409329 | 0.626158117 | 1.276678829 | 0.537922604 |
| SNHG25 | 0.874662231 | 0.553713791 | 1.38164162 | 0.565899014 |
| CASC15 | 1.064717057 | 0.658700317 | 1.720998733 | 0.797987081 |
| NR2F2-AS1 | 0.982733639 | 0.62413779 | 1.547359286 | 0.940057868 |
| LPP-AS2 | 1.317866957 | 0.572254564 | 3.034966303 | 0.516654679 |
| AC060766.7 | 1.062710243 | 0.617371974 | 1.829291106 | 0.826263318 |
| NR2F1-AS1 | 1.073304677 | 0.802666986 | 1.435194109 | 0.633223754 |
| AL049555.1 | 1.201357926 | 0.875089528 | 1.64927224 | 0.256507939 |
| HCP5 | 1.162382365 | 0.851275914 | 1.58718547 | 0.343741196 |
| AL391422.4 | 1.158674439 | 0.705312845 | 1.903448187 | 0.560895595 |
| AP000695.1 | 1.203104467 | 0.788438702 | 1.835856553 | 0.391138387 |
| LINC00857 | 1.386957461 | 0.98372891 | 1.955468604 | 0.061990385 |
| AC104083.1 | 0.876477918 | 0.66181779 | 1.160762905 | 0.357644377 |
| LINC00426 | 0.98966854 | 0.694918335 | 1.409437295 | 0.954092808 |
| MIR497HG | 0.757671262 | 0.496385746 | 1.156491188 | 0.198396403 |
| AC133644.2 | 0.989897241 | 0.691934193 | 1.416170149 | 0.955680903 |
| AC146944.2 | 0.908547322 | 0.605630735 | 1.362972829 | 0.643019966 |
| PCED1B-AS1 | 0.981249202 | 0.777240001 | 1.23880654 | 0.873531888 |
| AL133415.1 | 1.397408882 | 0.842427564 | 2.318005331 | 0.195007184 |
| AC135068.8 | 0.901381534 | 0.646656612 | 1.256445314 | 0.540053335 |
| LINC01140 | 0.959727077 | 0.710590477 | 1.296212225 | 0.788651143 |
| AC018755.4 | 0.731097004 | 0.492164319 | 1.086025153 | 0.120843599 |
| LINC00861 | 0.849074807 | 0.587145547 | 1.227852328 | 0.384678454 |
| MIR155HG | 0.692817503 | 0.37612564 | 1.276158923 | 0.238985243 |
| AC011899.2 | 0.752107082 | 0.427806031 | 1.322246583 | 0.322363802 |
| LINC01480 | 0.818719709 | 0.562424293 | 1.191808337 | 0.296469827 |
| AC139530.1 | 0.941718584 | 0.696733768 | 1.272844712 | 0.696081955 |
| LINC01857 | 0.876966067 | 0.672862814 | 1.142981104 | 0.331409584 |
| PCAT19 | 0.880428084 | 0.555702439 | 1.394907699 | 0.587548184 |
| AC093278.2 | 0.698283648 | 0.405540988 | 1.202344689 | 0.195209677 |
| AC243960.1 | 0.841936117 | 0.600863113 | 1.179730308 | 0.317486944 |
| MIR99AHG | 0.960610518 | 0.664311051 | 1.389067011 | 0.830892953 |
| LINC01638 | 1.066492937 | 0.731416644 | 1.555074245 | 0.737966205 |
| C22orf34 | 0.707712653 | 0.448212293 | 1.117455294 | 0.137957259 |
| AL583785.1 | 0.896660513 | 0.656498441 | 1.224679337 | 0.492867725 |
| AC022706.1 | 1.031348363 | 0.59989662 | 1.773104582 | 0.911102309 |
| AC011481.1 | 0.749031905 | 0.463835497 | 1.209585722 | 0.23728633 |
| FOXD2-AS1 | 0.861845415 | 0.568254815 | 1.307120503 | 0.484149249 |
| LBX2-AS1 | 1.048250675 | 0.756377446 | 1.452752835 | 0.7771642 |
| AC021016.2 | 0.599949731 | 0.298092476 | 1.20747657 | 0.152240843 |
| ITGA9-AS1 | 0.694748667 | 0.448542333 | 1.076098451 | 0.102798898 |
